#  A Novel Hypoxia-related lncRNA Risk Score Model for Prognosis Evaluation of Clear Cell Renal Cell Carcinoma

**DOI:** 10.2174/1386207326666230606152615

**Published:** 2024-09-25

**Authors:** Fu Liu, Xinyuan Li, Xiang Zhou, Hang Tong, Zili Hu, Xuesong Bai, Xin Gou

**Affiliations:** 1 Department of Urology, The First People's Hospital of Ziyang, Sichuan, China;; 2 Department of Urology, The First Affiliated Hospital of Chongqing Medical University, Chongqing, China;; 3 Chongqing Key Laboratory of Molecular Oncology and Epigenetics, Chongqing, China;; 4 Department of Urology, The Second Affiliated Hospital of Chongqing Medical University, Chongqing, China

**Keywords:** ccRCC, hypoxia-related genes, long non-coding RNAs, prognosis model, GSEA, renal cell carcinoma

## Abstract

**Background:**

Renal cell carcinoma is the most common aggressive tumor of the genitourinary system. The main pathological subtype is clear cell renal cell carcinoma (ccRCC), and its treatment options are very limited. Therefore, identifying specific biomarkers of ccRCC is of great significance for diagnosis and prognosis.

**Methods:**

First, we obtained transcriptome data and clinical data of 611 patients with renal clear cell carcinoma to analyze the relationship between hypoxia-related lncRNAs and overall survival (OS). We screened hypoxia-related lncRNAs through Pearson correlation and Cox regression analysis. Univariate and multivariate regression analysis were applied to assess survival-related risk factors. According to the median risk score, patients were divided into two groups. Next, a nomogram map was built, and GSEA was used for gene function annotation. RT-qPCR, Western Blot, and Flow Cytometry were used to determine the role of SNHG19 in RCC cells.

**Results:**

By analyzing the co-expression of hypoxia genes and lncRNAs, 310 hypoxia-related genes were obtained. Four sHRlncRs (AC011445.2, PTOV1-AS2, AP004609.3, and SNHG19) with the highest prognostic values were included in the group to construct the HRRS model. The high-risk group had a shorter OS than the low-risk group. HRRS was considered to be an independent prognostic factor and associated with OS. The two groups showed different pathways in GSEA. Experiments showed that SNHG19 plays essential roles in the autophagy and apoptosis of RCC cells.

**Conclusion:**

We constructed and validated a hypoxia-related lncRNA model for ccRCC patients. This study also provides new biomarkers for the poor prognosis of ccRCC patients.

## 1 INTRODUCTION

Renal cell carcinoma (RCC) is the most common genitourinary cancer in adults, and 270,000 new cases occur yearly worldwide and account for 2-3% of all malignant tumors [1]. According to different molecular genetic features, RCC was divided into different histopathologic types. Accounting for 70-80% of RCC, clear cell renal cell carcinoma (ccRCC) constitutes the main histopathological subtype [2]. Patients with ccRCC are usually diagnosed as advanced and have a poor prognosis because of its insidious onset and partly due to the lack of typical symptoms, effective diagnosis, and treatment methods [3]. ccRCC is not sensitive to chemotherapy and radiotherapy, especially in the advanced ccRCC [4]. The biological behavior of ccRCC is dynamic and complex [5]. There is still a lack of effective biomarkers to evaluate the prognosis of patients with ccRCC. Consequently, it is essential to search for a robust prognostic predictor with ccRCC [6].

Tumor biomarkers are substances or processes that can be used for prognosis, risk evaluation, and therapeutic responses. Long noncoding RNAs (lncRNAs) are seen as new prognostic biomarkers for many cancers, such as hepatocellular carcinoma [7], gastric cancer [8], lung cancer [9] and pancreatic cancer [10]. Hypoxia is an important feature of the tumor microenvironment, is associated with poor prognosis, and promotes tumor cell invasion, proliferation, angiogenesis, metastasis, and treatment resistance [11]. Some studies have reported that under hypoxic conditions, lncRNAs promote oral squamous cell carcinoma invasiveness *via* lncRNA HAS2-AS1 [12]. In pancreatic cancer, lncRNAs promote cancer metastasis *via* epithelial-mesenchymal transition [13]. Based on the important roles of lncRNAs in tumors, many studies have focused on potentially predicting progression and prognosis. lncR-ZNF180-2 is significantly expressed in advanced RCC [14]. To our knowledge, although many studies have found that lncRNAs are differentially expressed in many cancers, the role of lncRNAs in predicting ccRCC remains unclear [15]. Therefore, identifying promising prognostic markers based on hypoxia-related lncRNA expression is eagerly anticipated.

This research was designed to provide further insight into the clinical potency prognosis estimation based on hypoxia-related lncRNAs in ccRCC. HR-lncR genes with different expression levels were analyzed, and a risk score model was established to predict the poor prognosis of ccRCC patients. Then, we further calculated correlations between them and OS. Additionally, we constructed a nomogram to assess the clinical significance and validated the model. We also identified underlying biological processes and molecular mechanisms through GSEA. Finally, we built a more personalized accuracy prediction model for ccRCC, which provides a novel perspective. We also found that SNHG19 could promote autophagy and inhibit apoptosis in ccRCC cells.

## 2 MATERIALS AND METHODS

### 2.1 Patient Data Acquisition

We downloaded the transcriptome RNA sequencing of ccRCC samples and clinical information from The Cancer Genome Atlas (TCGA, https://portal.gdc.cancer.gov/), which included 532 ccRCC tissues and 79 nontumor tissues. Patients were excluded (n = 17) if their survival time was less than 30 days because these patients may have died due to other factors, such as hemorrhage or infection (Supplementary Table **S1**). We collected the raw data, which were updated on January 11, 2021, and performed further analysis. We used a merged script in the Perl language (http://www.perl.org/) to combine the RNA-seq results into a matrix file. Next, the Ensembl database GRCh38.p13 (http://asia.ensem bl.org/) was applied to convert the Ensembl IDs of related genes into a matrix of gene symbols. The study was carried out in compliance with all relevant guidelines and regulations.

### 2.2 Cell Culture and Transfection

The commonly used 786-O, CakI, RCC-23, and RCC-JF cell lines of ccRCC and human normal renal cell line HK-2 were purchased from American Type Culture Collection (ATCC, Manassas, Virginia, USA). The cells were cultured in DMEM medium with 10% fetal bovine serum and penicillin (100 IU/mL) and streptomycin (100 mg/mL) in a hypoxic environment (0.5% O_2_, 5% CO_2_, 94.5% N_2_). The culture medium was changed every 1-3 days. The si-RNAs were constructed and purchased from GenePharma company (Shanghai, China), and the sequences are listed in Table **S2**. 786-O was transfected with si-SNHG19 using LipofectamineTM 2000 (Invitrogen, Carlsbad, CA, USA) following the instructions of the manufacturer.

### 2.3 Hypoxia‐related lncRNAs

We downloaded the hypoxia-related gene set from the Molecular Signatures Database v4.2 (Cellular response to hypoxia, M5466; Genes known to be induced by hypoxia M641, M11033, and M10508; https://www.gsea-msigdb.org/gsea/msigdb/index.jsp) and obtained 310 hypoxia-related genes. Coexpression analysis was applied to analyze the association between the hypoxia-related genes and lncRNA expression in patients with ccRCC. HR-lncRs were identified by the criteria of *P* value < 0.001 and |cor| > 0.8.

### 2.4 Survival-related HR-lncRs

We used univariate and multivariate Cox analysis to identify hypoxia-related lncRNAs whose expression levels were significantly correlated (*P* < 0.05) with the OS of patients with ccRCC. We also used the hazard ratio (HR) to classify sHRlncRs into deleterious and protective groups. Subsequently, the candidate sHRlncRs were selected for the following study.

### 2.5 Establishment of the Hypoxia-related Risk Score Model (HRRS)

We used multivariate analysis to verify the reliability of the sHRlncRs and used the integrated sHRlncRs as an independent prognostic indicator to develop the HRRS (*p*<0.05 Table **2**). We used the differential expression of sHRlncRs to perform a risk score model, which used the median risk score as the cutoff point. The ccRCC patients were divided into high- and low-risk groups. The expression data multiplied by Cox regression coefficients established the HRRS model. The formula was as follows: Risk Score = (AC011445.2* (0.6152809)) + (PTOV1-AS2*(0.2626787)) + (AP004609.3*(-1.370513126)) + (SNHG19*(0.296 05893 5)). We evaluated the HRRS value in various subtypes of ccRCC patients.

### 2.6 Establishment and Validation of the Nomogram

A graphic nomogram based on the HR-lncRNA signature with clinicopathologic features was developed using R software (version 4.0.3) to predict the probable 3-year and 5-year survival of ccRCC patients. The time-varying ROC curve and calibration chart were used to verify the prediction accuracy of the nomogram. The clinical results predicted by the nomogram are displayed on the x-axis and y-axis, in which the 45-degree dashed line represents the ideal prediction.

### 2.7 Validation of the Hypoxia Prognosis Model in Predicting Survival

Stratified survival analysis was applied to check the accuracy of the prognostic signature in predicting the survival outcome of the patient. Kaplan–Meier curves were used for evaluating whether the risk score was an independent factor of other clinical variables, such as age, sex, grade, T stage, M stage, N stage, and stage, in determining the prognosis of ccRCC patients.

### 2.8 Gene Set Enrichment Analysis (GSEA)

The genome-wide expression profiles of the ccRCC patients were subjected to gene set enrichment analysis (GSEA version 4.0.3, http://www.gsea-msigdb.org/gsea/index.jsp) to determine the genes that were differentially expressed between patients in the high- and low-risk groups. In the current study, the gene sets of “c2.cp. Kegg.v7.2.symbols.gmt (Curated)” from the Molecular Signatures Database were analyzed.

### 2.9 Real-Time Quantitative PCR

Total RNA was extracted from cells with TRIzol (Takara Biotechnology Co., China) according to the instructions. The total RNA was reverse transcribed into complementary DNA (cDNA) using the Reverse Transcriptase PCR kit (Takara Biotechnology Co, China). Real-time PCR was performed using the SYBR-Green method according to the manufacturer’s instructions. GAPDH was used as the internal control and the primer sequences are shown in Table **S3**.

### 2.10 Western Blot Analysis

Cells were collected and lysed in ice-cold RIPA lysis buffer (Beyotime, Shanghai, China) containing PMSF and phosphatase inhibitor for 30 min. The BCA method was used to determine the concentration of each sample, and protein loading treatment and quantitative protein samples was used. The samples were electrophoresed with polyacrylamide gel and then transferred to a PVDF membrane. The PVDF membrane carrying protein was sealed with 5% skim milk for 2 h and incubated with primary antibody at 4°C overnight. The second antibody was incubated the next day, and the number of protein samples on the PVDF membrane was detected by chemical radiography. Primary antibodies list: P62(#23214, Cell Signaling Technology, Inc.), LC3I-II(#83506, Cell Signaling Technology, Inc.), β-actin (20536-1-AP, Proteintech Group, lnc.)

### 2.11 Flow Cytometry

Cells were suspended in PBS buffer at 48 h post-trans-fection, fixed in 75% ethanol at 4°C overnight, and stained with propidium iodide (PI) at room temperature for 30 min. For the analysis of cell apoptosis, harvested cells were suspended in PBS and stained using an Annexin V-fluorescein isothiocyanate/PI kit (cat. no. 556570, FITC Annexin V Apoptosis Detection kit II, BD Biosciences). The stained cells were analyzed by flow cytometry.

### 2.12 Statistical Analysis

The data were processed through the PERL programming language (version 5.30.2, http://www.perl.org). Pearson correlation analysis and Cox regression analysis were used to identify the HR-lncRNAs. The OS difference of patients in the low- and high-risk groups was evaluated by Kaplan-Meier curve analysis. Univariate and multivariate Cox regression analysis were performed to identify the independent prognostic factors for patients with ccRCC. All statistical analysis were performed using R software (version 4.0.3, https://www.r-project.org/) and GraphPad Prism 8 (GraphPad Software Inc, La Jolla, CA). *P*<0.05 was considered to indicate significance.

## 3 RESULTS

### 3.1 Acquisition of HR-lncRs

We downloaded the transcriptome data and clinical data from ccRCC patients in the TCGA database. The patients with OS<30 days were excluded. In total, 611 ccRCC samples were analyzed, including 539 ccRCC samples and 72 normal tissues. Transcriptome data were converted from Ensembl IDs to gene names and were divided into lncRNAs and mRNAs. We also obtained 310 hypoxia-related genes (M641, M5466, M11033, and M10508) from MSigDB. Finally, 14 hypoxia-related lncRNAs (| cor| > 0.8, *P* value < 0.001) were identified through coexpression analysis.

### 3.2 The Relevance of HR-lncRs and Prognosis

**Fig. (1):**
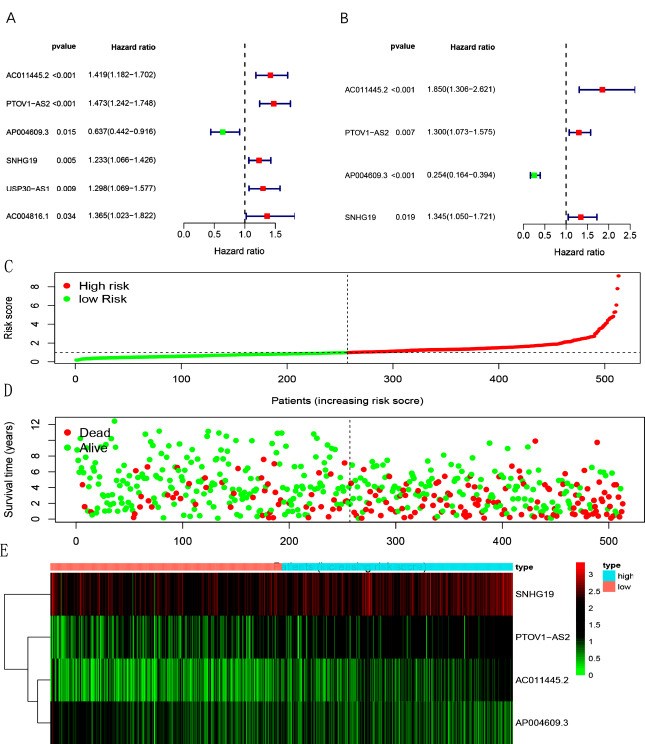
Survival-related values and hypoxia-related lncRNA model of hypoxia-related lncRNAs. Forest plot of hazard ratios showing the survival-related values of hypoxia-related lncRNAs by univariate Cox regression (**A**) and multivariate Cox regression models (**B**). Red parts represent upregulated hypoxia-related lncRNAs, and green parts represent downregulated hypoxia-related lncRNAs. Distribution of hypoxia-related lncRNAs in the high- and low-risk groups (**C**). Survival status between the high- and low-risk groups (**D**). Heatmap showing the expression profile of sHRlncRs (**E**).

**Fig. (2):**
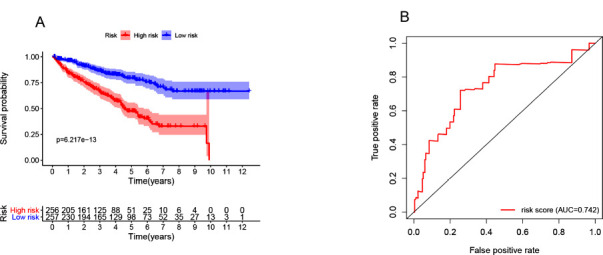
Survival curve of ccRCC patients and Receiver operating characteristic (ROC) curve. Kaplan-Meier survival curve analysis showed that the OS of ccRCC patients in the high-risk group was significantly shorter than that of ccRCC patients in the low-risk group (**A**). The ROC curve indicates the prognostic value of independent prognostic factors (**B**).

We identified HR-lncRs through univariate and multivariate Cox regression that were associated with prognosis. Then, we got 4 hypoxia-related lncRNAs: AC011445.2, PTOV1-AS2, AP004609.3, and SNHG19. The relationships between these HR-lncRs and prognosis are intuitively shown in the forest map (Fig. **1A** and **B**). These 4 HR-lncRs were constituted into the risk evaluation model. Based on the intermediate risk score, the ccRCC samples were divided into high- and low-risk groups (Fig. **1C**). Patients with higher risk scores had a higher mortality rate than patients with lower risk scores (Fig. **1D**). As the risk score increased, the expression levels of SNHG19, PTOV1-AS2, and AC011445.2 were elevated, while that of AP004609.3 was decreased (Fig. **1E**). Patients with lower risk scores had longer survival times (Fig. **2A**). This indicates that the risk score has a prognostic value.

### 3.3 The HR-lncRNA Signature is an Independent Prognostic Factor

Univariate and multivariate regression analyses were used to determine the risk factors for assessment. The univariate analysis revealed that the overall survival rate was related to age, stage, grade, T stage, N stage, M stage, and HRRS (*P*<0.05). However, multivariate analysis showed an increase for only the HRRS, and age was significantly related to the overall survival rate (Table **1**). The area under the ROC curve represented the accuracy of the entire model. The area under the ROC curve of HRRS was 0.742 (Fig. **2B**). These results showed that HRRS can be a prognostic factor in patients with ccRCC.

### 3.4 Evaluation of the Prognostic Prediction Nomogram, Including the HR-lncRNA Prognostic Signature Risk Score

**Fig. (3):**
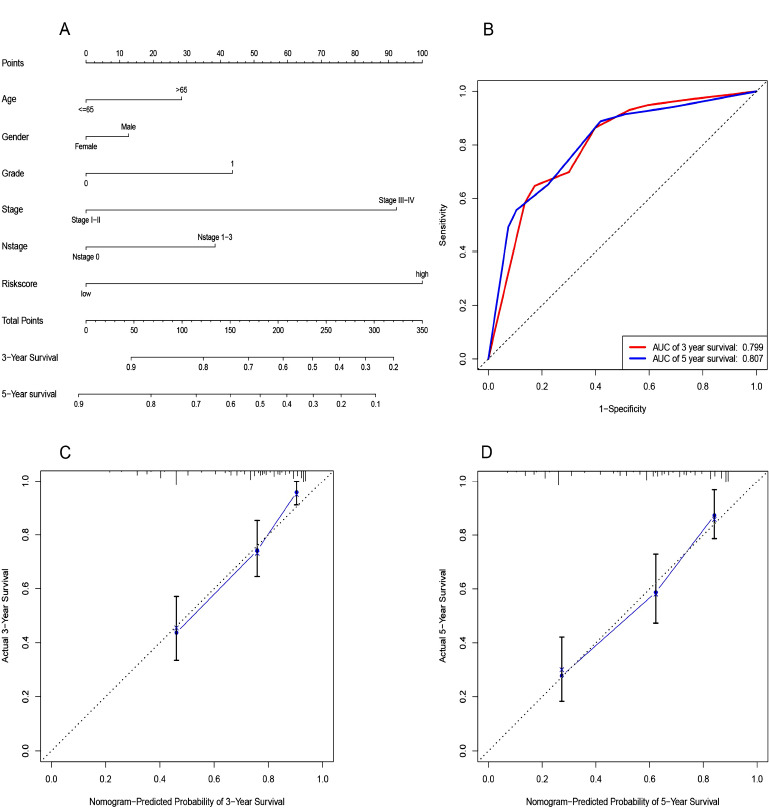
A nomogram was established for predicting 3- and 5-year OS. A nomogram combining the 4-lncRNA signature and clinical factors was used to predict the 3-year and 5-year OS of ccRCC patients in the TCGA dataset (**A**). Time-dependent ROC curve for the predictive accuracy of the risk model for 3- and 5-year OS (**B**). Calibration curve of the nomogram for the prediction of 3- and 5-year OS (**C, D**).

Nomograms are tools commonly used by clinicians to accurately predict survival time for a patient by calculating the nomogram score based on the points assigned for each prognostic factor included in the nomogram [16]. To accurately estimate the 3-year and 5-year survival probabilities, we established a nomogram by risk score calculated from the HR-lncRNA prognostic signature and other clinicopathological factors (age, sex, grade, stage, and N stage, Fig. **3A**). The AUCs of 3- and 5-year survival were 0.799 and 0.807, respectively (Fig. **3B**). The calibration curve analysis showed that compared with the reference line, the actual and predicted survival rates at 3 and 5 years were consistent (Fig. **3C** and **D**). These results demonstrated that the nomogram using the HR-lncRNA prognostic signature risk scores was reliable and accurate.

### 3.5 The Hypoxia Prognosis Model Effectively Predicts the Survival of ccRCC Patients

**Fig. (4):**
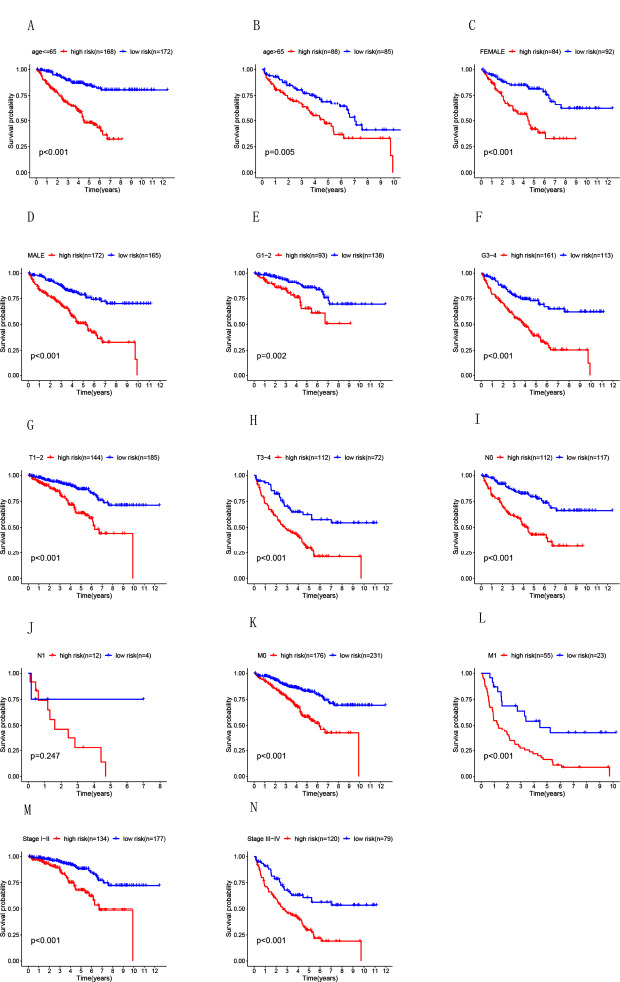
The value of the Kaplan-Meier curve for the prognosis of the patient’s risk score divided by the clinical characteristics Age (**A, B**), sex (**C, D**), G stage (**E, F**), T stage (**G, H**), N stage (**I, J**), M stage (**K, L**) and stage (**M, N**).

Patients with renal clear cell carcinoma were stratified according to age (≤ 65 and > 65), sex (male and female), G stage (G1-2 and G3-4), T stage (T1-2 and T3-4), N stage (N0 and N1-3), M stage (M0 and M1), and stage (stages I-II and III-IV) to verify the predictive performance of the risk scores. The subgroup analysis results indicated that the overall survival rate (OS) of the low-risk group was significantly higher than that of the high-risk group (Fig. **4**). However, there were no significant differences in the N1-3 groups (*p* = 0.247), which may be due to the small sample size.

### 3.6 Gene Set Enrichment Analysis

**Fig. (5):**
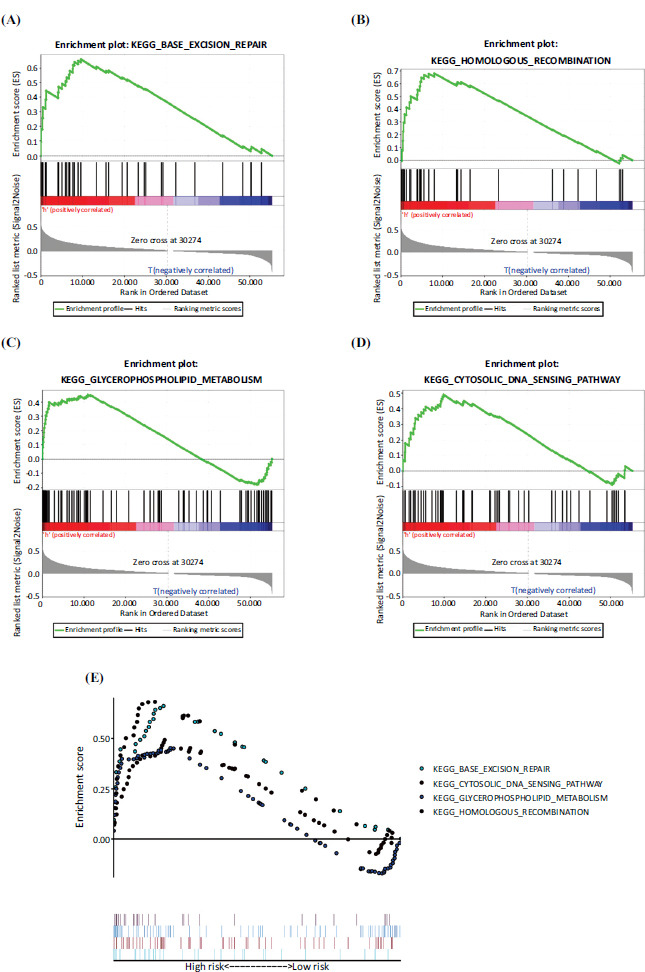
According to the prognostic characteristics of hypoxia-related lncRNAs, gene set enrichment analysis (GSEA) was performed on high-risk and low-risk ccRCC patients. The GSEA results show significant enrichment of hypoxia-related signaling pathways in high-risk ccRCC patients (**A-D**). The main KEGG signaling pathways in the high-risk ccRCC patients (**E**).

We used GSEA to study the potential molecular mechanisms of lncRNA signaling related to hypoxia in the progression of ccRCC. The results showed base excision repair, homologous recombination, glycerophospholipid metabolism, and cytosolic DNA sensing pathways (Fig. **5A**-**D**). A study revealed that 27-32% of RCC tissues contain mutations in homologous recombination genes [17]. Another study revealed that homologous recombination, base excision repair, and glycerophospholipid metabolism as differentially enriched in ccRCC [18]. It suggested that those pathways are very important in ccRCC. The main KEGG pathways in the high-risk GSEA are shown in Fig. (**5E**).

### 3.7 Suppression of SNHG19 Inhibits Autophagy and Promotes Tumor Cell Apoptosis

As shown in Fig. (**1E**), the SNHG19 expression was higher. SNHG19 was selected for further experiments. To explore the role of SNHG19 in ccRCC, we performed functional *in vitro* experiments. The results of RT-qPCR showed that the expression level of SNHG19 in ccRCC cell lines (786-O, CAKI, RCC-JF) was remarkably higher than that in the renal tubular epithelial cell line (HK2).786-O cell was chosen for subsequent research, in which SNHG19 is the most highly expressed (Fig. **1A**). We verified the knockdown efficiency of three SNHG19-siRNA(SNHG19-siRNA1, SNHG19-siRNA2, and SNHG19-siRNA3)in 786-O cell, and SNHG19-siRNA3 was the most effective siRNA (Fig. **1B**). Therefore, SNHG19-siRNA3 was selected for all subsequent experiments.

**Fig. (6):**
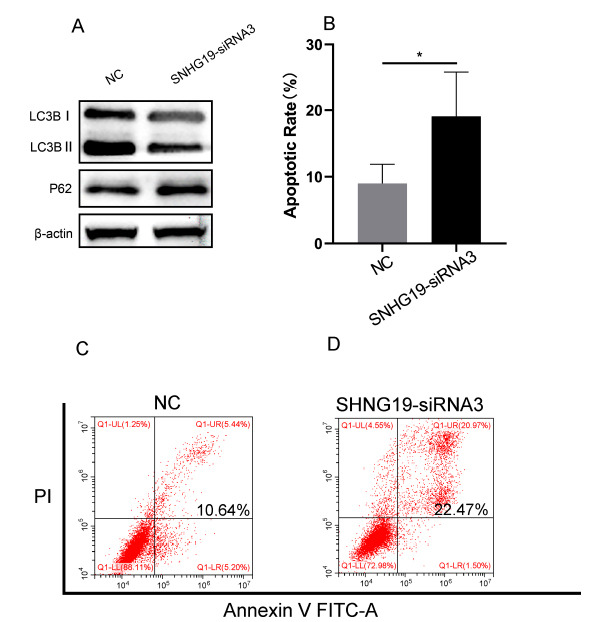
SNHG19 plays essential role in autophagy and apoptosis of 786-O. Western blot analysis of the expression levels of LC3 II/LC3 I, P62 (**A**); Apoptosis was analyzed by flow cytometry (**B-D**). (NC: Negative Control, *P<0.05).

After SNHG19 expression was silenced, the LC3B-II/I ratio was significantly decreased, while the P62 expression level was significantly increased (Fig. **6A**), as detected by western blotting. the apoptotic index of 786-O was significantly increased after SNHG19 expression was silenced, as detected by flow cytometry (*P*<0.05) (Fig. **6B**-**D**). These findings indicate that SNHG19 could promote autophagy and inhibit apoptosis.

## 4 DISCUSSION

RCC is one of the most common types of urological tumors, with a high recurrence rate and a mortality rate of more than 40% [19, 20], especially when metastasis occurs. Almost 30% of patients undergoing radical or partial nephrectomy experience recurrence or metastasis, which also means a poor prognosis [21]. Although innovative and multimodal treatment strategies, including immunotherapy and targeted therapy, have provided many ccRCC patients with more novel options and have prolonged their survival time, the remaining patients have not yet received satisfactory treatment [2, 22, 23].

Hypoxia is a microenvironmental feature of many tumors and is related to poor prognosis and treatment failure. Therefore, finding robust prognostic biomarkers remains an urgent challenge. With the rapid development of bioinformatics technology, new prognostic markers in ccRCC can be identified. Because of their improvement in predictive accuracy in comparison to standard benchmarks, researchers have recently paid more attention to lncRNA-based biomarkers [24-26].

lncRNAs are a newly discovered type of noncoding RNA molecule involved in regulating tumor cell development, differentiation, proliferation, and apoptosis [27]. Hence, they are potential biomarkers that can predict tumor risk and survival outcomes. In our study, we first obtained 611 ccRCC patients from TCGA. Univariate Cox regression analysis was used to investigate the expression of hypoxia-related lncRNAs, and we identified 14 hypoxia-related lncRNAs that significantly correlated with OS. Furthermore, the top 4 hypoxia-related lncRNAs-namely, AC011445.2, PTOV1-AS2, AP004609.3, and SNHG19-were applied to establish a prognostic signature based on their performance in the multivariate Cox regression analysis. Utilizing a risk-scoring model, we divided ccRCC patients into low-risk and high-risk groups. Due to molecular heterogeneity, traditional clinicopathological parameters are insufficient for accurately predicting the survival of patients with ccRCC, and we further verified the predictive value of these four sHRlncRs through multivariate analysis. This lncRNA signature was also related to poor OS in ccRCC patients of different subgroups, especially the age, grade, stage, T stage, N stage, and M stage subgroups. This signature was an independent risk factor for OS.

Nomograms can be used for personalized risk assessment according to the characteristics of patients and are widely used to evaluate tumor prognosis [16]. Then, we constructed a nomogram to evaluate the OS of ccRCC patients. Compared with the traditional stage and grade system, our nomogram was more accurate and had higher clinical value.

We observed that our hypoxia‐related lncRNA signature was involved in cell base excision repair, homologous recombination, glycerophospholipid metabolism, and the cytosolic DNA sensing pathway. Many studies have reported the importance of these pathways in cancer. Base excision repair (BER) removes approximately 40,000 endogenous lesions per human cell every day. The recognized cause of cancer is the accumulation of genetic damage leading to genomic instability. BER plays a vital role in cancer prevention. This topic has been covered recently in some excellent reviews [28-33]. Homologous recombination is a DNA repair pathway, and defective DNA repair is a common sign of cancer. Since homologous recombination-deficient cells are sensitive to poly(ADP-ribose) polymerase (PARP) inhibitors, they have attracted clinical interest [34]. Abnormal glycerophospholipid (GPL) metabolism is a common metabolic hallmark of cancer and is associated with tumor progression, which is represented by phosphatidylethanolamine (PE) and phosphatidylcholine (PC) [35]. Some studies have shown elevated PC levels in most cancers, including the brain [36, 37], breast [38, 39], ovarian [40], cervical [41], endometrial [42], and prostate [43] cancers. Cytosolic DNA sensing, involving the cyclic GMP-AMP synthase-stimulator of interferon genes (cGAS-STING) pathway, may play an important role in antitumor immunity [44]. Li *et al*. reported that the cGAS-STING pathway plays a crucial role in maintaining the antitumor immunity of stem cell-like T cells [45]. Further research, the downregulation of SNHG19 by siRNA treatment inhibits autophagy and promotes tumor cell apoptosis. This may provide little evidence for the poor survival in RCC patients.

Although our research shows that hypoxia-related lncRNA signatures are stable, some limitations remain. First, our findings must be further validated in other independent cohorts to verify the accuracy of the hypoxia-related lncRNA prognostic signature. Second, our study is based on a single cohort study of 611 patients from an international database and has been verified internally. Moreover, there is little research on our studied hypoxia-related lncRNAs. The mechanism of action of lncRNAs related to hypoxia in ccRCC needs further experimental verification.

## CONCLUSION

We constructed a prognostic signature comprising 4 hypoxia-related lncRNAs to predict the OS of ccRCC and verified this signature. Furthermore, the characteristics of the 4 lncRNAs related to hypoxia that we established can predict the clinical value of this predictive nomogram model. It can better predict the prognosis of ccRCC patients than the traditional stage and grade system. We hope that this signature will provide new references for the current prediction of ccRCC prognosis and provide new ideas for hypoxia-related research and treatment strategies.

## Figures and Tables

**Table 1:** The results of multivariate Cox regression coefficients.

**Gene**	**coefficients**	**HR**	**HR.95L**	**HR.95H**	***P* value**
AC011445.2	0.615281	1.850176	1.306058	2.62098	0.000535
PTOV1-AS2	0.262679	1.300409	1.073449	1.575354	0.00727
AP004609.3	-1.37051	0.253977	0.163772	0.393865	9.24E-10
SNHG19	0.296059	1.344549	1.050457	1.720978	0.018732

**Note: ** HR: Hazard Ratio.

**Table 2:** Univariate and multivariate analysis of ccRCC.

**Variables**	**Univariate analysis**				**Multivariate analysis**			
	**HR**	**HR.95L**	**HR.95H**	***P* value**	**HR**	**HR.95L**	**HR.95H**	***P* value**
Age	1.020541	1.002458	1.038951	0.025806	1.028036	1.007621	1.048865	0.006897
Gender	1.077652	0.703577	1.650615	0.731011	1.362737	0.858767	2.162463	0.188951
Grade	2.144173	1.614471	2.847668	1.37E-07	1.362619	0.972588	1.90906	0.072112
Stage	1.853064	1.525999	2.250228	4.79E-10	1.473277	0.86717	2.503019	0.151878
T	1.878724	1.482565	2.380743	1.80E-07	0.901389	0.545072	1.490633	0.685833
M	4.207377	2.714666	6.520885	1.30E-10	1.631829	0.709407	3.753648	0.249247
N	3.145137	1.62416	6.090466	0.000678	1.547833	0.752324	3.184515	0.2353
Risk Score	1.466671	1.314895	1.635966	6.34E-12	1.244418	1.08854	1.422618	0.001363

## Data Availability

Authors can provide all of the datasets analyzed during the study on reasonable request.

## LIST OF ABBREVIATIONS

GSEA: Gene Set Enrichment Analysis
HRGs: Hypoxia-Related Genes
HR-lncRs: Hypoxia-related Long Non-coding RNAs
HRRS: Hypoxia-related Risk Score
lncRNAs: Long Non-coding RNAs
OS: Overall Survival
PCA: Principal Component Analysis
RCC: Renal Cell Carcinoma
sHRlncRs: Survival-related HR-lncRs
TCGA: The Cancer Genome Atlas
