# Supplementary material for: A Novel Hypoxia-related lncRNA Risk Score Model for Prognosis Evaluation of Clear Cell Renal Cell Carcinoma
Source: Comb Chem High Throughput Screen. 2024 Sep 25;28(15):2589–600. doi: 10.2174/1386207326666230606152615 (PMC12824858; doi:10.2174/1386207326666230606152615)
Supplement: Supplementary file 1 [file CCHTS-28-15-2589_SD1.pdf]

Supplementary Material

A Novel Hypoxia-related lncRNA Risk Score Model for Prognosis Evaluation of Clear Cell Renal Cell Carcinoma

Fu Liu[1,2], Xinyuan Li[2], Xiang Zhou[2,3], Hang Tong[2], Zili Hu[4], Xuesong Bai[2,*] and Xin Gou[2,*]

[1]*Department of Urology, The First People's Hospital of Ziyang, Sichuan, China;* [2]*Department of Urology, The First Affiliated Hospital of Chongqing Medical University, Chongqing, China;* [3]*Chongqing Key Laboratory of Molecular Oncology and Epigenetics, Chongqing, China;* [4]*Department of Urology, The Second Affiliated Hospital of Chongqing Medical University, Chongqing, China*

Supplementary table S1. The summary of clinical characteristics of patients with ccRCC

Characteristics	TCGA
Age(years)	
≤65	352
>65	185
Gender	
Male	346
Female	191
Grade	
G1	14
G2	230
G3	207
G4	78
Stage	
SI	269
SII	57
SIII	125
SIV	83
T-stage	
T1	275
T2	69
T3	182
T4	11
N-stage	
N0	240
N1	17
M-stage	
M0	426
M1	79

Supplementary Table S2. Sequences of SNHG19-siRNA.

Small interfering RNA
si-SNHG19-1#:
5′ -CAUGAUUUCUGAAATTUGUCAUUAUUCUCAAUGAGTTGAACA-3′ .
si-SNHG19-2#:
5′ -UGAGGTTACUCACUUUACCUCTTUAAAGUGAGUUAUUAAAUA-3′ .
si-SNHG19-3#:
5′ -UACAAAGAATTUUGGGAGTTCUCCCUUCUAUCACUUAGUGAG-3′ .

Supplementary Table S3. Primer sequences used for RT-qPCR.

Gene	Forward (5' to 3')	Reverse (5' to 3')
SNHG19	GCTACGATCTTGGGACGAACT	GGCCTGAGATTCGGGAAGAAA
GAPDH	GGTGAAGGTCGGAGTCAACG	CAAAGTTGTCATGGATGHACC
